# Ictal Onset Signatures Predict Favorable Outcomes of Laser Thermal Ablation for Mesial Temporal Lobe Epilepsy

**DOI:** 10.3389/fneur.2020.595454

**Published:** 2020-10-15

**Authors:** Naoir Zaher, Alexandra Urban, Arun Antony, Cheryl Plummer, Anto Bagić, R. Mark Richardson, Vasileios Kokkinos

**Affiliations:** ^1^Department of Neurology, University of Pittsburgh, Pittsburgh, PA, United States; ^2^University of Pittsburgh Comprehensive Epilepsy Center, Pittsburgh, PA, United States; ^3^Department of Neurosurgery, Massachusetts General Hospital, Boston, MA, United States; ^4^Harvard Medical School, Boston, MA, United States

**Keywords:** epilepsy, epilepsy surgery, temporal lobe epilepsy, laser abaltion, seizure onset pattern

## Abstract

**Background:** Laser interstitial thermal therapy (LiTT) has emerged as a minimally invasive option for surgical treatment of refractory epilepsy. However, LiTT of the mesial temporal (MT) structures is still inferior to anterior temporal lobectomy (ATL) in terms of postoperative outcome. In this pilot study, we identify intracranial EEG (iEEG) biomarkers that distinguish patients with favorable outcome from those with poor outcome after MT LiTT.

**Methods:** We performed a retrospective review of 9 adult refractory epilepsy patients who underwent stereotactic electroencephalography (sEEG) followed by LiTT of MT structures. Their iEEG was retrospectively reviewed in both time and frequency domains.

**Results:** In the time-domain, the presence of sustained 14–30 Hz in MT electrodes coupled with its absence from extra-MT electrodes at ictal onset was highly correlated with favorable outcomes, whereas the appearance of sustained 14–30 Hz or >30 Hz activity in extra-MT sites was negatively correlated to favorable outcomes. In the frequency domain, a declining spectral phase, beginning at the high frequency range (>14 Hz) at ictal onset and following a smooth progressive decline toward lower frequencies as the seizure further evolved, was positively correlated with improved outcomes. On the contrary, low frequency (<14 Hz) patterns and “crescendo-decrescendo” patterns with an early increasing frequency component at ictal onset that reaches the high-beta and low gamma bands before decreasing smoothly, were both correlated with poor surgical outcomes.

**Conclusions:** This pilot study demonstrates the first evidence that iEEG analysis can provide neurophysiological markers for successful MT LiTT and therefore we strongly advocate for systematic sEEG investigations before offering MT LiTT to TLE and MTLE patients.

## Introduction

Despite the introduction of many anti-seizure medications over the last decades, surgical treatment remains the most effective way of achieving seizure freedom for patients with refractory temporal lobe epilepsy (TLE) (1, 2). Anterior temporal lobectomy (ATL) has repeatedly and consistently demonstrated superior postoperative seizure control, with 60–80% of patients achieving seizure freedom at 1–2 years post-resection, and almost 50% of patients experiencing prolonged seizure freedom up to 10 years (3, 4). However, there is a subgroup of patients with drug-resistant epilepsy that either does not fulfill the candidacy criteria for (5) or are uncomfortable with (6) resective surgery.

Since its approval in the US in 2010, laser interstitial thermal therapy (LiTT) (7), often simply referred to as laser ablation, has emerged as a minimally invasive option for surgical treatment of refractory epilepsy in both adult (8, 9) and pediatric populations (8). Primarily due to its ability to reach deep foci with minimal disruption of the surrounding neuronal tissue, laser ablation has become the preferred method of treatment in selected cases. Patients with mesial TLE (MTLE) constitute a significantly large target group that benefit from this surgical approach, especially if selective amygdalohippocampectomy is proposed (10, 11) or if patients are above 50 years of age (12). As a result of the minimally invasive nature of the procedure and minimal disruption of tissue adjacent to the ablation target, patients have a shorter post-operative recovery duration (13) and reduced post-operative cognitive impairments (11, 14–16) compared to resective surgery.

Seizure freedom rates following laser ablation in MTLE have been reported to approach that of temporal lobectomy, with seizure freedom rates reaching up to 69% at 12 months (17) and 54% at 26 months (13). However, meta-analysis recently demonstrated that after LiTT only 58% of patients with TLE and 66% of MTLE patients achieve seizure freedom (18). It is also generally accepted that patients with MTLE benefit from sEEG monitoring to confirm MT seizure onset, and that the presence of extra-temporal or bi-temporal onset predicts persistence of seizures following resection. A recent study demonstrated that by confirming the MT onset with sEEG, patients without hippocampal sclerosis can achieve seizure freedom rates similar to patients with MRI-diagnosed hippocampal sclerosis following selective laser amygdalohippocampectomy (19). However, the use of sEEG in patients undergoing LiTT therapy is not systematic, therefore most published studies on this patient population do not report iEEG data. In a previously published series of 13 patients, only one out of four TLE patients without hippocampal sclerosis (HS) was seizure free following ablation; interestingly it was the only patient with sEEG-confirmed seizure onset (13). In another study, 3/4 of TLE patients without hippocampal sclerosis on MRI and with seizure onset localized to the hippocampus by means of sEEG became seizure free (11). None of these studies was complemented by iEEG analysis in an effort to establish neurophysiological predictive factors of surgical outcome.

Overall, laser ablation of the mesial temporal (MT) structures is still inferior to ATL in terms of postoperative outcome, as the subgroup of MTLE patients that fail to achieve seizure freedom or improved seizure control following hippocampal laser ablation is larger than the respective group treated with ATL (8). Several factors across different stages of both the electro-clinico-anatomical assessment of the seizure onset zone may contribute to poor outcomes, as well as the surgical procedure itself. However, the focal nature of LiTT treatment provides a unique opportunity to correlate MTLE patient outcomes with anatomically restricted intracranial neurophysiology when intracranial monitoring is performed prior to LiTT. This opportunity is not available in the context of wide “en block” ATL resections that encompass the removal of several distinct structures with independent epileptogenic potential. In this pilot study, we focus exclusively on identifying intracranial electroencephalographic (iEEG) signatures that cluster patients with suspected MT pathology in those that most likely will experience a favorable outcome and those with a risk for poor outcome following laser ablation of the MT structures.

## Materials and Methods

### Patients

**Figure 1:**
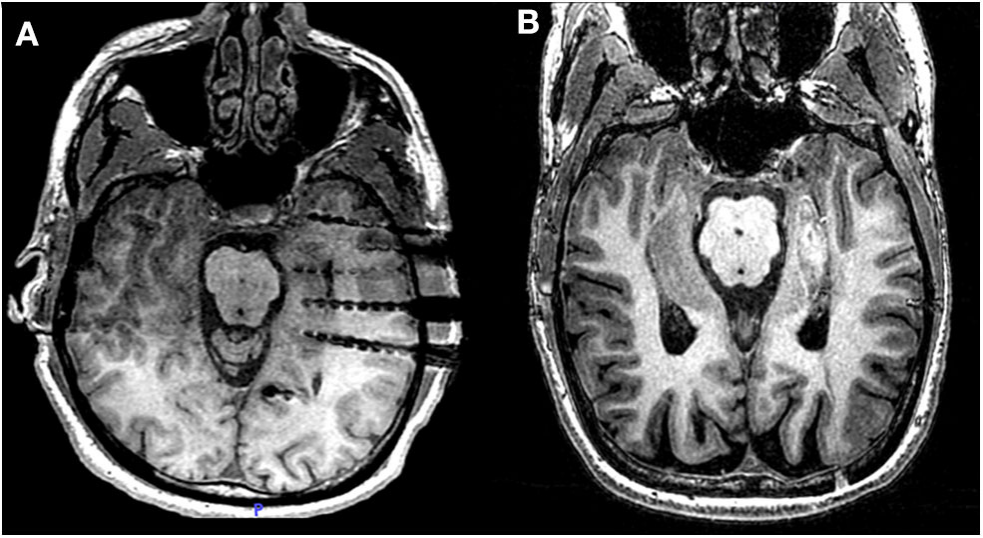
Laser ablation after sEEG investigation. **(A)** Post-operative MRI of patient 2 showing three electrodes covering the head, the body and the tail of the hippocampus. From each electrode lead, two contacts are recording from hippocampal parenchyma. **(B)** Post-ablation MRI of the same patient, showing the extent of the lesion across the MT longitudinal axis.

We performed a retrospective review of adult refractory focal epilepsy patients admitted for presurgical evaluation at the University of Pittsburgh Medical Center between 2014 and 2019. Subjects were included if they met the following criteria: (1) they underwent stereotactic electroencephalography (sEEG) prior to ablation, as non-invasive data could not support a solid hypothesis of MTLE for them; (2) sEEG electrode coverage included at least one lead in the hippocampus (Figure 1A); (3) MT LiTT was decided by a multidisciplinary patient management conference of experts after taking into account sEEG findings, in conjunction with accompanying presurgical evidence and patient preferences; (4) MT ablation focused on the hippocampus and extended through the amygdala and the entorhinal cortex (we will refer to the cluster of these structures as the MT complex, Figure 1B); (5) surgical treatment did not include a combination of LiTT with other therapy types (e.g., resection, responsive neurostimulation); (6) they had at least 12 months of post-operative follow-up. The ictal iEEG segments from each patient's intracranial monitoring admission were reviewed retrospectively. The study was approved by the University of Pittsburgh Institutional Review Board (IRB).

### Data and Analysis

iEEG signals were recorded using a 128-channel Xltek digital system (Natus Medical Incorporated, Pleasanton, CA), at a sampling rate of 1 kHz. A board-certified epileptologist (N.Z.) and an epilepsy surgery neurophysiologist (V.K.) reviewed the iEEGs of the selected patients, and selected ictal iEEG segments from all clinical seizures that contributed to the decision for MT LiTT. The seizure onset was determined by the first change of the iEEG signal in the context of a sustained rhythmic discharge (20, 21) followed by or concurrently associated with clinical signs on simultaneous video (22). Pre-ictal spiking was treated as interictal hyperexcitability and was not included in the ictal phase. Ictal iEEG signature evaluation was performed without filters and focused in the first 5 s after the seizure onset (22) and the emergence of delta (1–3 Hz), theta (4–7 Hz), low beta (8–13 Hz), high beta (14–30 Hz) and gamma (>30 Hz) frequency bands was registered for each event. Ictal activity for each frequency band was further categorized in terms of its sustainability, that is, its potential to maintain rhythmicity for more than 3 s, allowing for variances in amplitude, in contrast to activity that appears in an intermittent and irregular fashion (e.g., rhythmic blocks of 1 up to 2 s or less). The 3 s threshold was determined as the 60% portion of the 5 s analysis window. The dominant ictal onset features for each patient, as a cumulative neurophysiological description of all the recorded ictal onsets, were decided by consensus. The temporal order of ictal manifestations within the 5 s window were not accounted for in our biomarker-oriented study. sEEG electrode channels were in turn categorized in terms of (a) implication in the seizure onset, and (b) inclusion in the area of the ablated MT complex. The volume/extent of ablation was retrospectively reviewed in all post-surgical MRIs and sufficient ablation volume within the pre-defined limits of the MT complex was confirmed in our patient cohort.

Time-frequency analysis was performed for each ictal segment by means of the Fast Fourier Transform (FFT) of a 1024-point window and a 1000-point overlap for frequencies from 0.05 to 40 Hz at a step of 0.05 Hz with 1/f normalization adjustment using Matlab (The Mathworks, Natic, MA, USA). The power dimension of the spectrum was represented in absolute values and displayed in a linear hot-colored grayscale. For each patient, a single grand average was created derived from the individual 3D time-frequency power tables of all ictal iEEGs and all channels residing in the parenchyma of the MT complex.

Evaluation of post-operative seizure control compared to the pre-operative baseline was done by retrospective review of the patients' electronic medical records. The documentation of quantitative (frequency and duration of seizures) and qualitative (severity of seizures) features was used to formulate outcome assessments. Post-operative seizure control assessments were scored by means of the Engel outcome scale (23).

Two-sided Fisher exact test was used to assess statistical significance (*p* < 0.05) between the emergence of each iEEG pattern for each frequency band with outcome levels. For statistical analysis, iEEG ictal onset evaluations were reduced to binary representations, denoting the appearance and absence of each frequency band for each patient. A reduced binary representation was also used for favorable (Engel Class I and II) and poor outcomes (Engel Class III and IV).

## Results

Out of a pool of 27 patients that underwent MT laser ablation, nine (9) patients (mean age 37 ± 9.5, two males) were subjected to pre-operative intracranial EEG monitoring. None of these patients had prior intracranial investigations or surgeries. Seven patients lacked any structural abnormalities on MRI, and two were found to fulfill the criteria of hippocampal sclerosis. A total of 74 seizures were analyzed (mean 8.2 ± 7.8). The average number of recording contacts residing in the hippocampus, amygdala and uncus was 4.3 ± 1.1 (total of 39 electrodes). Ablation was performed on left MT structures in four patients, and on the right side in the rest. Average follow up after surgery was 22.7 months, ranging from 16 to 29 months. A total of 4 patients (44%) achieved Engel class I outcome; two patients became free of disabling seizures (Engel class IA), one patient continued to experience rare auras (Engel class IB) and one patient reported a single habitual seizure following withdrawal of antiepileptic medication (Engel class ID). The rest of the patients (56%) failed to achieve sufficient seizure control after laser ablation and were categorized with either III or IV Engel class outcomes (Table 1).

**Table 1:** Patient demographic and clinical data.

**Patient**	**Gender**	**Age**	**MRI MT findings**	**PET/iSPECT MT findings**	**Neuropsych evaluation**	**Number of clinical seizures**	**Number of sEEG contacts in the MT Complex**	**Ablated MT Lobe**	**Non-ablated site: range of rhythmic activity at onset (Hz)**	**Ablated site: range of rhythmic activity at onset (Hz)**	**Follow-up (Engel Classification)**
1	F	26	Normal	LMTa	LMTd	1	5	RMT	**14–30**, **>30**	4–7, **8–13**, **14–30**, >30	IIIA at 16 months
2	F	48	Normal	LMTa	BMTd	3	6	LMT	n/a	4–7, **8–13**	IVB at 19 months
3	F	36	Normal	LMTa	LMTd	12	4	LMT	**14–30**, **>30**	4–7, **14–30**, **>30**	IVB at 29 months
4	F	38	RMTa	LMTa	RMTd	5	4	RMT	n/a	**8–13**, **14–30**	IA at 19 months
5	F	51	RMTa	RMTa	BMTd	10	4	RMT	n/a	**8–13**, **14–30**	ID at 27 months
6	F	24	Normal	Normal	RMTd	3	5	RMT	n/a	**8–13**, **14–30**	IB at 26 months
7	M	40	Normal	n/a	LMTd	27	2	LMT	**>30**	1–3, 4–7	IV at 21 months
8	F	42	Normal	Normal	RMTd	6	5	RMT	**14–30**	**14–30**	IIIA at 25 months
9	M	28	Normal	n/a	LMTd	7	4	LMT	n/a	**8–13**, **14–30**	IA at 16 months

*The ictal iEEG patterns observed sustained in the specified band limits for more than 3 s in the 5-s window of the seizure onset are in bold fonts; transient non-sustained rhythmic activity observed in the respective sites appears in plain fonts. RMT, right mesio-temporal; LMT, left mesio-temporal; L/RMTa, left/right mesio-temporal abnormality; L/R/BMTd, left/right/bilateral mesio-temporal dysfunction; n/a, not available or no rhythmic activity observed*.

### Time-Domain Ictal Onset Signatures

We examined the predominant frequency of ictal activity at onset, both in the ablation site and extra-mesial non-ablated sites by visually evaluating the recordings in the time domain (findings are summarized in Table 1). We found that the presence of sustained 8–13 or 14–30 Hz activity alone was not an indicator of favorable outcome (*p* = 0.16 and 0.44, respectively). However, the exclusive presence of sustained 14–30 Hz activity in the MT structures, with its concurrent absence from extra-MT structures, was highly correlated with good outcomes (*p* < 0.01). Similarly, the exclusive presence of 8–13 Hz in the MT structures was correlated with a good outcome, although to a lesser degree (*p* = 0.04). On the other hand, the appearance of low-frequency components in the MT complex at the seizure onset (1–3 Hz or 4–7 Hz bands) was correlated with poor postoperative seizure control (*p* = 0.04). The manifestation of >30 Hz activity in the MT complex and in extra-MT structures was not predictive of favorable seizure control outcomes (*p* = 0.43 and 0.16, respectively). The appearance of sustained 14–30 Hz or more than 30 Hz activity in extra-MT non-ablated sites at the seizure onset was negatively correlated to favorable outcomes (*p* = 0.04). All patients with concurrent appearance of 14–30 Hz in both MT and extra-MT sites (patients 1, 3, and 8) had poor surgical outcomes.

### Frequency-Domain Ictal Onset Signatures

**Figure 2:**
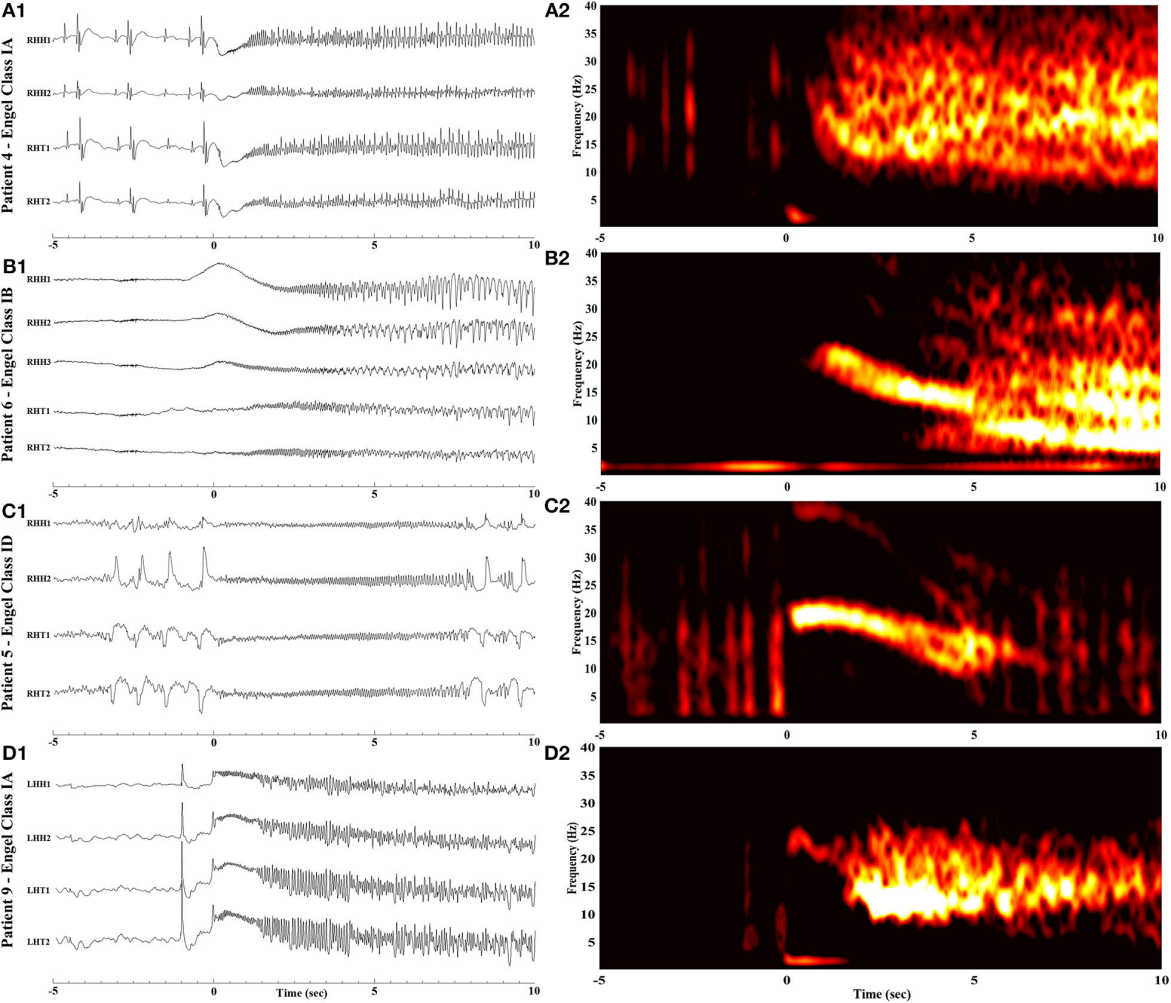
Raw iEEG (left, **A**_**1**_**-D**_**1**_) from the MT contacts and respective time-frequency analysis (right, **A**_**2**_**-D**_**2**_) of seizure onsets in patients with favorable outcome. **(A**_**1**_**,A**_**2**_**)** Patient 4 with Engel Class IA outcome. **(B**_**1**_**,B**_**2**_**)** Patient 6 with Engel Class IB outcome. **(C**_**1**_**,C**_**2**_**)** Patient 5 with Engel Class ID outcome. **(D**_**1**_**,D**_**2**_**)** Patient 9 with Engel Class IA outcome. Notice that for all four patients, ictal frequency progressively declines following ictal onset (time = 0).

**Figure 3:**
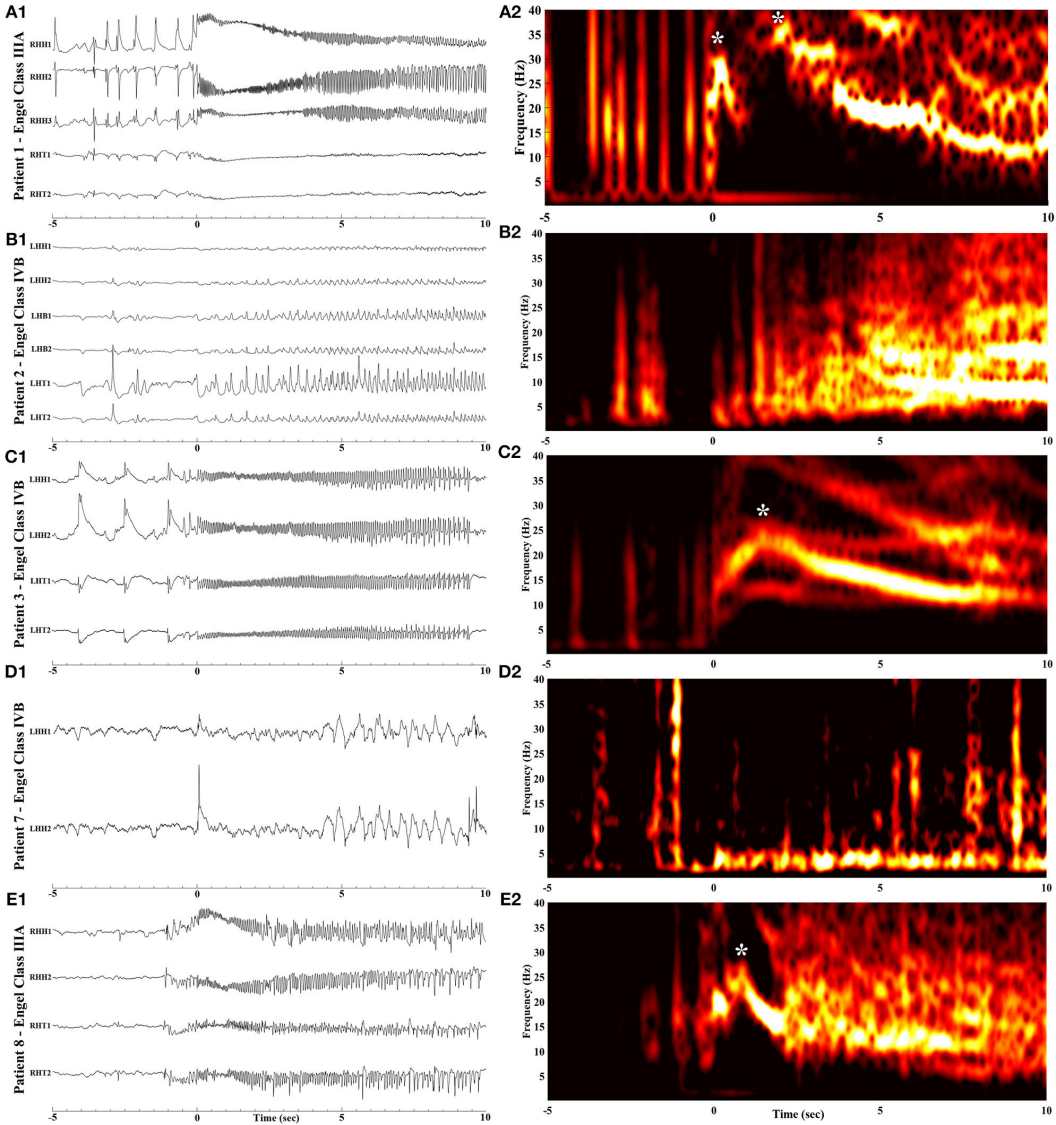
Raw iEEG (left, **A**_**1**_**-E**_**1**_) from the MT contacts and respective time-frequency analysis (right, **A**_**2**_**-E**_**2**_) of seizure onsets in patients with poor outcome. **(A**_**1**_**,A**_**2**_**)** Patient 1 with Engel Class IIIA outcome. **(B**_**1**_**,B**_**2**_**)** Patient 2 with Engel Class IVB outcome. **(C**_**1**_**,C**_**2**_**)** Patient 3 with Engel Class IVB outcome. **(D**_**1**_**,D**_**2**_**)** Patient 7 with Engel Class IVB outcome. **(E**_**1**_**,E**_**2**_**)** Patient 8 with Engel Class IIIA outcome. Notice the rise in frequency content with a peak indicated by asterisks, following ictal onset (at time = 0).

The time-frequency analysis plots of the ictal iEEG recorded from the MT complex contacts demonstrated recognizable and systematic spectral features in patients with favorable outcome. Specifically, we observed a single smoothly declining spectral phase, beginning at the high frequency range at ictal onset and following a smooth progressive decline toward lower frequencies as the seizure further evolved (Figure 2). This spectral pattern was not observed in patients who failed to achieve seizure freedom. In this category of patients, we observed two main patterns (Figure 3). The first pattern comprised of two phases: an initial component of increasing frequency at ictal onset that reached the high-beta and low gamma bands before decreasing smoothly, thereby creating a distinct “crescendo-decrescendo” pattern (the crescendo's peak is indicated by asterisks in Figures 3A_2_,C_2_,E_2_). The second pattern was a low-frequency onset profile, that may (Figure 3B_2_) or may not (Figure 3D_2_) recruit higher frequencies as the seizure evolves. Both patterns were associated with poor post-surgical outcomes.

## Discussion

Although there have been many efforts to identify ictal onset patterns and link them to outcome following surgery for MTLE (24–29), all prior studies evaluated outcome following gross surgical resections, mainly ATLs. ATLs are not limited to the structure being recorded during the invasive investigation phase but extend beyond the implanted structures, thereby confounding the interpretation of the ictal onset pattern and its link to outcome. In contrast, laser ablation is a highly selective procedure, thereby allowing for more confident correlations. Our pilot study focused on the laser ablation of a specific epileptogenic target (the MT complex, comprising of the hippocampus, the amygdala at the junction of the hippocampus and surrounding entorhinal cortex), which allows us to draw highly specific conclusions regarding the effect this selective intervention has on the seizure onset zone, and in turn make a reliable correlation between ictal onset patterns and outcome.

In this pilot work we identified iEEG biomarkers in the seizure initiation zone that can be used to predict outcome following MT laser ablation. Favorable outcome was associated with a sustained 14–30 Hz activity present exclusively in the MT region at ictal onset, in the absence of extra-MT rhythmic activity, suggesting that this specific pattern of ictal onset was arising from the “genuine” seizure-onset zone. In contrast, the presence of activity in the 14–30 Hz range concurrently in both MT and extra-MT sites, as well as in extra-MT structures alone, may be suggestive of an epileptogenic zone that extends beyond the MT region. Our results support the observation that the presence of low voltage fast activity (LVFA)—generally defined as EEG activity exceeding 14 Hz—remains the most important prognostic factor when it comes to EEG onset patterns (30–32). However, the benefit for clinical evaluations can be minimal, as LVFA from both the ictal onset zone and the propagating zones can be indistinguishable (27). This can be very challenging especially when the available spatial coverage is limited. Through our pilot study we made a potentially useful observation in separating ictal onset patterns between patients who fail laser ablation in the MT structures and those who achieve seizure freedom. Patients with poor post-operative seizure control manifested a spectral pattern that comprised of two phases: an initial increase of frequency content followed by a subsequent reduction (a “crescendo-decrescendo” spectral pattern). In contrast, patients with good outcome manifested a LVFA profile that progressively shifted to lower frequencies following the ictal onset. The underlying neuronal mechanism of the two patterns is not clear. It has been hypothesized that the primary source of ictal activity is a migrating ictal wavefront (33). In this context, it is possible that the observed initial increase in frequency following the onset in patients with poor postoperative seizure control represents a traveling ictal wavefront reaching the MT complex from a nearby structure. In other words, the crescendo part of the bi-phasic spectral pattern can be interpreted as a local MT reactive element to epileptic activity initiating in a neighboring region, suggesting that MT-only ablation is likely to fail and that ATL is more likely to benefit the patient. This distinction in ictal onset patterns is particularly important for clinical decision-making. To our knowledge this is the first time a distinction is made at this level, partly due to the fact that time-frequency analysis is not routinely performed in clinical practice and is usually not a part of iEEG record reviewing. Although recently a significant effort has begun to identify spectral signatures that denote genuine ictal onsets (32), the material for that study was derived from various brain structures that underwent resection of variable extend, thereby not allowing for a finer link between spectral fingerprints corresponding to specific recording sites and seizure control outcome.

Our results are also consistent with published work on seizure onset zone EEG activity from animal models. Ictal-like discharge initiating with a 20–30 Hz oscillation in the acute model of temporal lobe ictogenesis has been shown in the isolated guinea pig brain (34), and similar fast activity at onset was also replicated in computational models (35). In a literature review of prior work on intracranial ictal onset patterns (20), authors found that, although their description in human studies varied widely, most studies showed that fast activity >14 Hz onset portends a good prognosis. Only one study reported good outcome with ictal onset cutoff frequency at >8 Hz (25), which is lower than most other published work including ours; however the study was done using epidural electrodes which may have affected the ability to record higher frequencies. Consequently, it is not surprising that the presence of 14–30 Hz activity at the ictal onset in non-ablated sites correlated with poor seizure control following LiTT since its presence would indicate a wide seizure-initiation network that extends beyond the MT complex.

Our pilot study was limited in terms of our patient cohort mainly because sEEG is not currently established as a standard procedure before LiTT intervention in most centers, resulting in the majority of LiTT-treated patients lacking iEEG data. This is mainly due to: (1) The patients' reluctance to undergo an invasive diagnostic procedure that requires prolonged hospitalization, and (2) The lack of iEEG-derived biomarkers with high specificity for laser ablation success that could drive decision-making for LiTT. The latter can prevent involved physicians from proposing a sEEG study for prognostic purposes to patients that prefer laser ablation over resective treatment options. Our work demonstrates the first evidence that iEEG analysis can provide biomarkers for successful MT LiTT and therefore we strongly advocate for systematic sEEG investigation and iEEG evaluation in both time and frequency domains before offering MT LiTT to TLE and MTLE patients. We also hope to inspire a multi-center study in order to reach a larger sample size and improve statistical power, as we acknowledge that iEEG is not the single indicator of post-operative outcome and that there are many variables that should be factored-in when evaluating the patient's odds of seizure freedom following any procedure. However, despite the fact that our small cohort discouraged us from performing multiple regression analysis among the full spectrum of presurgical prognostic factors to demonstrate our correlations, our pilot study, by taking advantage of the laser's selectivity, helped us discern neurophysiological indicators of proximity of the epileptogenic zone that differentiate surgical outcome groups. Finally, there are currently no universal quantitative guidelines for describing the neurophysiological features of ictal iEEG onsets. We performed our seizure analysis solely on descriptions of the iEEG pattern and used a reasonable evaluation system that is partially based on prior published work in the subject (20–22, 36).

By highlighting iEEG markers, our pilot work suggests that there is prognostic value in the use of sEEG in patients that are candidates for hippocampal LiTT therapy, and could be considered as a more systematic investigation in TLE cases with unclear MT involvement. We envision the use of such sEEG-derived neurophysiological biomarkers to better inform both physicians and patients on the expected outcome following LiTT.

## Data Availability Statement

The raw data supporting the conclusions of this article will be made available by the authors, without undue reservation.

## Ethics Statement

The studies involving human participants were reviewed and approved by University of Pittsburgh Institutional Review Board. The patients/participants provided their written informed consent to participate in this study.

## Author Contributions

VK and NZ conceptualized and designed the study. NZ, AU, AA, CP, AB, VK, and RMR contributed in the performance of presurgical exams, data acquisition, evaluation, hypothesis formation and decision-making for all TLE patients treated with laser ablation. RMR performed all laser ablation procedures. VK and NZ derived, organized, reviewed and analyzed in the time domain all the data relevant to this study. VK performed the time-frequency analysis and statistics. VK, NZ, and RMR compiled the manuscript. All authors reviewed the manuscript and agreed with its contents.

## Conflict of Interest

The authors declare that the research was conducted in the absence of any commercial or financial relationships that could be construed as a potential conflict of interest.

## Abbreviations

ATL: anterior temporal lobectomy
LiTT: laser interstitial thermal therapy
MTLE: mesial temporal lobe epilepsy
MT: mesial temporal
iEEG: intracranial electroencephalography
sEEG: stereotactic electroencephalography
LVFA: low voltage fast activity.
